# The Pentagonal‐Pyramidal Hexamethylbenzene Dication: Many Shades of Coordination Chemistry at Carbon

**DOI:** 10.1002/chem.201705812

**Published:** 2018-03-09

**Authors:** Johannes E. M. N. Klein, Remco W. A. Havenith, Gerald Knizia

**Affiliations:** ^1^ Molecular Inorganic Chemistry, Stratingh Institute for Chemistry, Faculty of Science and Engineering University of Groningen Nijenborgh 4 9747 AG Groningen The Netherlands; ^2^ Zernike Institute for Advanced Materials and Stratingh Institute for Chemistry University of Groningen Nijenborgh 4 9747 AG Groningen The Netherlands; ^3^ Ghent Quantum Chemistry Group, Department of Inorganic and Physical Chemistry Ghent University Krijgslaan 281 (S3) 9000 Gent Belgium; ^4^ Department of Chemistry Pennsylvania State University 401A Chemistry Bldg; University Park PA 16802 USA

**Keywords:** bond theory, carbon, coordination modes, density functional calculations, donor–acceptor systems

## Abstract

A recent report on the crystal structure of the pentagonal‐pyramidal hexamethylbenzene dication C_6_(CH_3_)_6_^2+^ by Malischewski and Seppelt [*Angew. Chem. Int. Ed*. **2017**, *56*, 368] confirmed the structural proposal made in the first report of this compound in 1973 by Hogeveen and Kwant [*Tetrahedron Lett*. **1973**, *14*, 1665]. The widespread attention that this compound quickly gained led us to reinvestigate its electronic structure. On the basis of intrinsic bond orbital analysis, effective oxidation state analysis, ring current analysis, and comparison with well‐established coordination complexes, it is demonstrated that the central carbon atom behaves like a transition metal. The central (apical) carbon atom, although best described as a highly Lewis‐acidic carbon atom coordinated with an anionic cyclopentadienyl ligand, is also capable of acting as an electron‐pair donor to a formal CH_3_^+^ group. The different roles of coordination chemistry are discussed.

## Introduction

**Figure 1:**
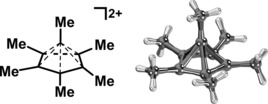
Structural depiction of the pentagonal‐pyramidal hexamethylbenzene dication C_6_(CH_3_)_6_^2+^ (left) and its crystal structure (right), as determined by Malischewski and Seppelt (CCDC‐1496 330).^1^

Malischewski and Seppelt recently reported the crystal structure of the pentagonal‐pyramidal hexamethylbenzene dication C_6_(CH_3_)_6_^2+^ (**I**) (Figure 1).^1^ This is unequivocal evidence for the structural assignment of this compound made in 1973 by Hogeveen and Kwant on the basis of spectroscopic studies.^2^

As an immediate response to the structural confirmation of **I**, scientific news outlets commented on this report^3^ with catchy titles such as “Six bonds to carbon: Confirmed”,3a and related claims that the established carbon bonding modes had been severely challenged, if not disproven. Although the Lewis structure depiction, as well as the depiction of the X‐ray structure, might suggest an unusual bonding scenario, we note that the original work by Malischewski and Seppelt^1^ did not claim any unusual bonding, that is, exceeding the common four‐bond limit for carbon. In fact, the authors suggested that the octet rule still stands, and that the bonding in **I** could be described as an interaction between a cyclopentadienyl cation and CH_3_C^+^.^1^

This description was already provided in the original spectroscopic study^2^ proposing **I**, and in early computational analyses.^4^ Intrigued by this compound, we decided to investigate its electronic structure. We here confirm that the compound can be understood with established bonding concepts from coordination chemistry, and that the usual four‐bond limit expected for carbon is not exceeded. However, contrary to the proposal of the original studies, we find that the compound is best described as a coordination complex with an anionic cyclopentadienyl ligand, a notion that was already hinted at in an early computational study of the related pentagonal‐pyramidal compound (CH)_6_^2+^.4b

## Results and Discussion

We began our exploration by optimizing the structure of **I** at the TPSS^5^‐D3(BJ)^6^/def2‐TZVP^7^ level of theory. This resulted in geometric parameters that agreed well with those determined experimentally (for full computational details see Supporting Information). On the basis of the obtained Kohn–Sham DFT wave function, we performed an intrinsic bond orbital (IBO)^8^ analysis of the electronic structure of **I**. We confirmed the absence of static correlation effects by using Grimme's test, to ascertain that our DFT treatment is appropriate (see Supporting Information for results).^9^ Under this condition, the IBOs, which pose a mathematically exact molecular orbital representation of the DFT wave function, provide a definitive and intuitively accessible description of the bonding: normally each IBO can be interpreted as an electron pair in a Lewis structure.^10^

**Figure 2:**
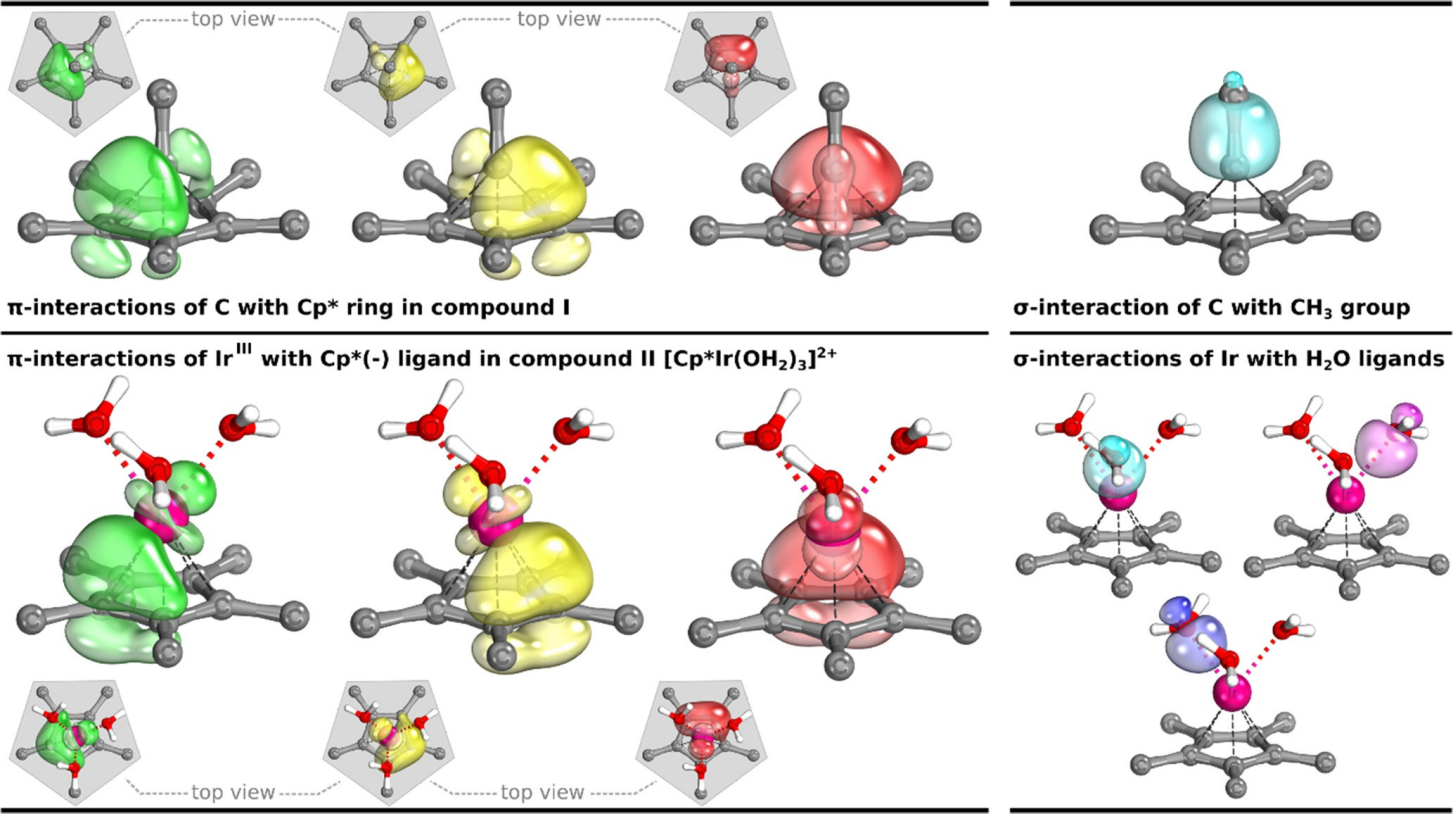
Comparison of the bonding interaction between the central atom and the Cp* ring in C_6_(CH_3_)_6_^2+^ (**I**) and [Cp*Ir(OH_2_)_3_]^2+^ (**II**). The latter is an undisputed Cp*(−) coordination complex. Depicted are isosurfaces of IBOs at the TPSS‐D3(BJ)/def2‐TZVP level of theory, each enclosing 80 % of the orbital electron's density. Hydrogen atoms bound to carbon are omitted for clarity. Visualized using IboView.10e, 11

For the C_6_(CH_3_)_6_^2+^ molecule **I**, we find four IBOs engaged in bonding with the apical carbon atom (Figure 2, top), indicating a total of four bonding interactions rather than six. Three of these can be identified as π‐bonding orbitals originating from the five‐membered ring, and one represents a σ‐bond from the directly bound CH_3_ group. The σ‐bond from the directly bound CH_3_ group reflects typical C−C bonding in hydrocarbons, albeit with strong polarization toward the apical carbon atom; this aspect will be discussed further below. In addition, each of the three π‐bond orbitals, which represent the bonding interaction between the apical carbon and the ring, is heavily polarized toward the apical carbon atom.

Curiously, rather than indicating the previously evoked Cp(+) moiety,^1^, ^2^, ^4^ the three π‐bond orbitals represent a bonding interaction strongly reminiscent of the bonding in transition metal complexes bearing cyclopentadienyl [Cp(−)] ligands, or more specifically, that found if a Cp(−) ligand is coordinated in η^5^ fashion and provides six electrons for coordination.^12^ Analogously to the C_6_(CH_3_)_6_^2+^ molecule **I**, pentamethylcyclopentadienyl [Cp*(−)] has been used as a more electron‐donating variant of this ligand for various transition metal complexes. So, should the apical carbon be described as a Lewis‐acidic transition metal, engaged in coordinative bonding to an aromatic π‐system?

**Figure 3:**
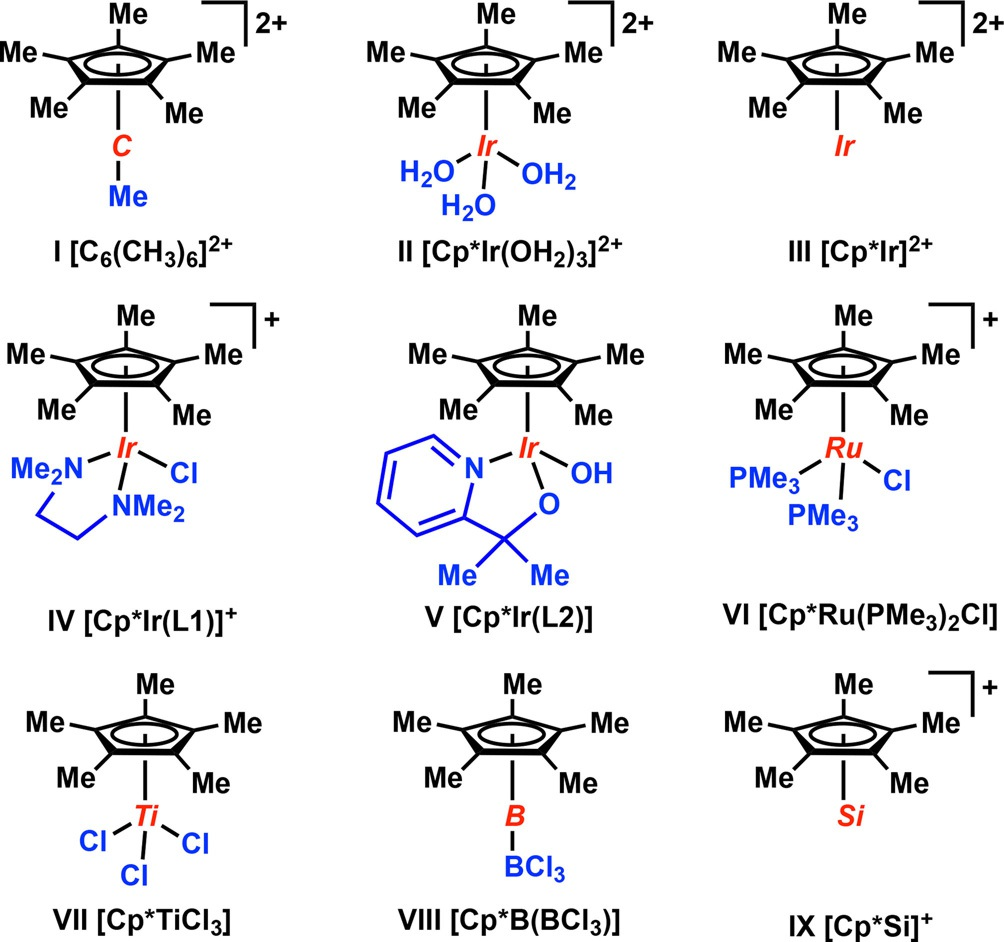
Summary of all studied compounds. For a list of relevant experimental references, see Supporting Information.

For a direct comparison, we selected a series of well‐defined Cp*‐containing transition metal complexes (**II**–**VII**) and two main‐group element compounds of similar composition (**VIII** and **IX**), as listed in Figure 3. This set of complexes allows us to directly compare the bonding between the Cp*(−) ligand and the transition metal or main‐group element to the bonding in **I**.

In all cases, we find three π‐bonding interactions between the ring and the apical coordinating atom. For complex **II**, IBOs are depicted in Figure 2 (bottom); the IBOs obtained for the rest of the complexes are given in the Supporting Information (Figure S2), as they proved to be very similar. As seen in Figure 2, the π‐bonding interaction of **II** shows a strong resemblance to the bonding observed in compound **I**. In addition to the strong resemblance between the IBOs of the Cp* moieties in all compounds, we also find good agreement between the averaged C−C bond lengths in the five‐membered ring. In C_6_(CH_3_)_6_^2+^, the C−C bond lengths between the carbon atoms of the Cp* ring are computed to be 1.451 Å. This compares well with the value of 1.442 Å determined experimentally,^1^ and lies midway between the calculated C−C bond lengths of the Cp*(−) complexes **II** to **IX**, ranging from 1.428 Å (in **VII**) to 1.465 Å (in **III**).

Apart from the crystal structure, Malischewski and Seppelt^1^ also reported calculations of the nucleus independent chemical shift (NCIS) of **I**, which indicate the presence of three‐dimensional aromaticity. Our bonding picture of **I** provides a straightforward explanation of this finding, as the π‐system of a Cp*(−) ring is aromatic according to the Hückel rules, and the Lewis‐acidity of the apical carbon atom in the +2 oxidation state draws this π‐system out of the plane, making it appear three‐dimensional.

**Figure 4:**
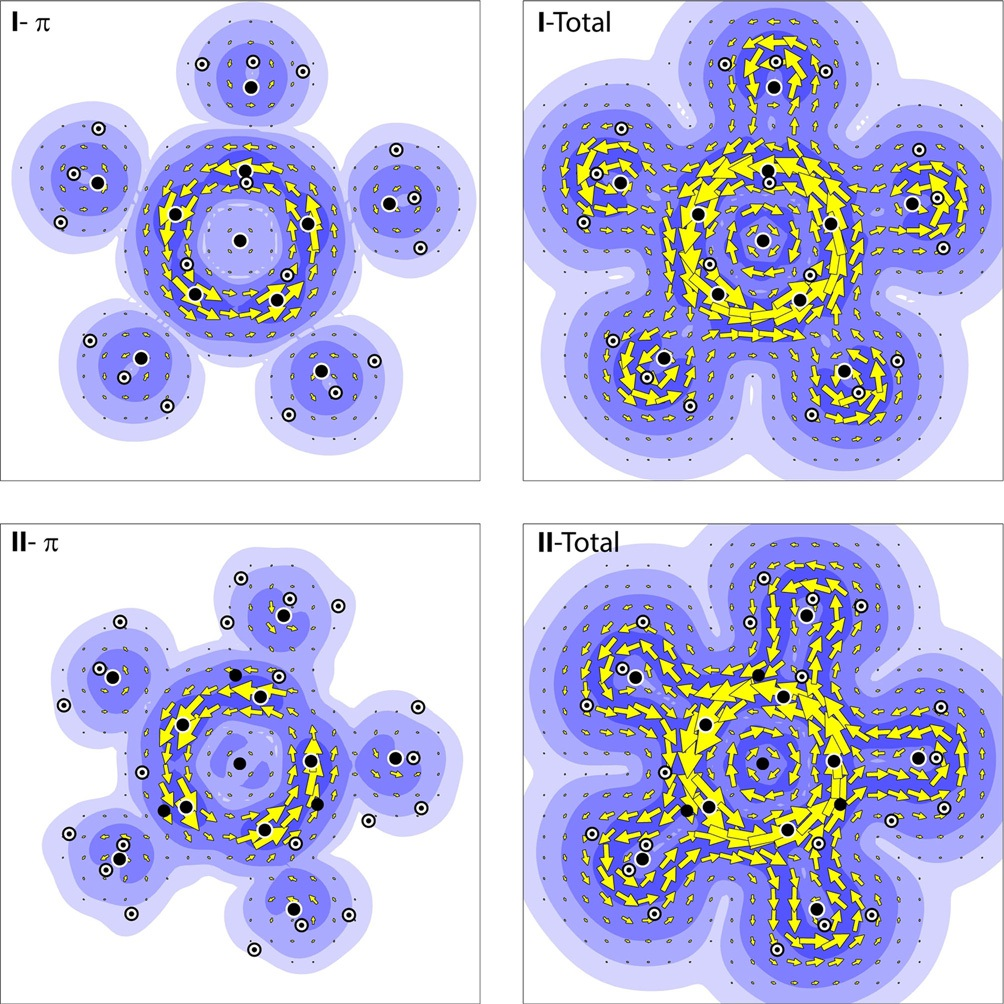
Plots of the contribution of the π‐like orbitals to the current density for **I** and **II** (**I**‐π and **II**‐π) and of the total induced current density (**I**‐Total and **II**‐Total).

As we compared the bonding in **I** to the bonding in transition metal complexes featuring Cp*(−) ligands, we decided to carry out ring current calculations for **I** and **II** at the DFT level of theory (for additional details see Supporting Information). As expected, we can clearly identify similar ring current patterns for both the contribution of the π‐like orbitals and the total induced current density in both **I** and **II** (Figure 4). A typical diatropic ring current is discernible, characteristic of an aromatic compound, and notably, does not differ much between a conventional coordination compound such as **II** and compound **I**.

For completeness, we computed intrinsic atomic orbital (IAO) partial charges of the complex fragments (see Supporting Information for details). For the C_6_(CH_3_)_6_^2+^ molecule **I**, the partial charge for the Cp* fragment is +1.563, which may seem inconsistent with an anionic Cp* ligand. However, partial charges are poor predictors of oxidation states.^13^ As an illustration, in the Ir complexes **III**, **II**, **IV**, and **V**, the Cp* fragment partial charges vary as +1.251, +0.617, −0.173, −0.195, all for formally anionic Cp*(−) ligands and identical Ir−Cp* binding. Therefore, the partial charge cannot be taken as indicative of the formal binding motive, as has been found in many other previous cases.^13^

To further address the role of oxidation states and validate our assignment as a Cp*(−) ligand coordinated to the apical carbon in compound **I**, we employ the recently introduced effective oxidation state (EOS) formalism of Salvador and co‐workers,^14^ which allows assignation of oxidation states on the basis of first‐principles wave functions in a well‐defined manner. This EOS analysis also confirms that the bonding in compound **I** is best described as an anionic Cp* ligand coordinated to carbon. As seen from Table 1, we find that the Cp* fragment is formally anionic for all the complexes. For the transition metal complexes **II**–**VII**, the EOS analysis also correctly identifies the established oxidation states of the transition metal centers.

**Table 1:** Computed effective oxidation states (EOS) of complexes **I**–**IX**.

Complex	EOS			R [%]^[b]^
	[Cp*]	[M]	[L]^[a]^	
**I** C_6_(CH_3_)_6_^2+^	−1	+2	+1	63.17 [61.77]
**II** [Cp*Ir(OH_2_)_3_]^2+^	−1	+3	0	61.30 [53.24]
**III** [Cp*Ir]^2+^	−1	+3	n.a.	57.63 [65.34]
**IV** [Cp*Ir(L1)]^+^	−1	+3	−1	70.28 [62.75]
**V** [Cp*Ir(L2)]	−1	+3	−2	78.09 [70.15]
**VI** [Cp*Ru(PCH_3_)_2_Cl]^+^	−1	+3	−1	77.77 [70.87]
**VII** [Cp*TiCl_3_]	−1	+4	−3	100.00 [91.90]
**VIII** [Cp*B−BCl_3_]	−1	+1	0	80.67 [61.51]
**IX** [Cp*Si]^+^	−1	+2	n.a.	100.00 [99.99]

[a] Group oxidation state for all ligands (except Cp*) bound to the [M] fragment. [b] Formal assignment reliability based on topological fuzzy Voronoi cells (TFVC) and based on intrinsic atomic orbitals (IAO). The latter values are given in brackets.

One interesting observation is made upon inspection of the oxidation states of the apical carbon atom and the attached methyl group in **I**. Here, we compute oxidation states of +2 and +1 for the apical carbon atom and the methyl group, respectively.

Although this bonding picture deviates from the proposed interaction between a cyclopentadienyl cation and CH_3_C^+^, it does agree with the bonding proposed for examples **VIII** and **IX**. For **VIII**, Frenking and co‐workers proposed that the boron atom bound to the Cp* fragment possesses a lone pair that coordinates to the BCl_3_ fragment.^15^ Note that similarities between carbon and boron have been discussed in the literature for compounds of this type.^16^ Similarly, for **IX**, a Si‐based lone pair was demonstrated.^17^ The monocationic all‐carbon compound analogue of **IX** was studied recently using computational methods by Pichierri,^18^ and was found to exhibit similar bonding properties, including the presence of a lone pair at carbon. Protonation of the apical carbon, for a variant lacking methyl substituents, leads to the pentagonal‐pyramidal C_6_H_6_ dication, which has been studied both experimentally and computationally.^19^ Notably, the idea of an anionic Cp fragment was put forward.19c In related blog posts by Rzepa,^20^ a close relative of **I**, in which the apical carbon is protonated and the CH_3_ groups on the five‐membered ring are retained, is studied. Again, a lone pair susceptible to protonation is discussed, and analyses include atoms in molecules (AIM)^21^ and electron localization function (ELF)^22^ investigations, leading to the suggestion by Rzepa of a hexacoordinate apical carbon, which he also describes as hexavalent, although he clearly states that these bonding interactions are not to be interpreted as conventional two‐electron sharing bonds, an idea that is discussed later in the context of helium bonds.^23^ The analyses are also in agreement with the notion that the octet rule is not violated. For the pentagonal‐pyramidal C_6_H_6_ dication and its relative, the interpretation of a carbon‐centered lone pair, which can be subject to protonation, is in line with our observation that the methyl group attached to the apical carbon atom is identified as cationic in the EOS analysis. Furthermore, a description of this type is in line with the polarization of the σ‐bond identified by the IBO analysis (Figure 2, top), with a partial charge distribution of the associated IBO between the apical carbon and the CH_3_ carbon of 1.139 and 0.860, respectively.

**Figure 5:**
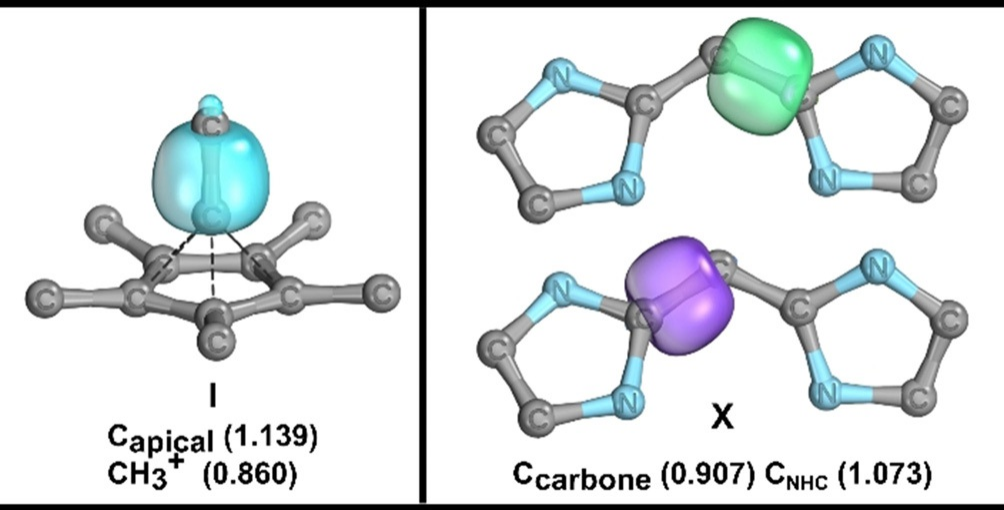
Comparison of the polarized σ‐bonds in **I** and **X**. IAO partial charges of the depicted IBOs are given. Visualized using IboView.10e, 11

Considering the EOS analysis, the comparison to the protonated congener [C_6_H_6_]^2+^, and the observation of noticeable polarization, one could describe this σ‐bond as a coordinative bond. Frenking and co‐workers have studied dative bonding for main‐group elements,^24^ including carbodicarbenes,^25^ extensively. For carbodicarbenes they suggest that the C−C σ‐bonds are best described as donor–acceptor/dative bonds.^25^ Although controversial, the use of arrows to indicate such C−C bonds is recommended by Frenking and co‐workers.^26^ For a direct comparison, we computed IBOs of the relevant σ‐bonds for carbodicarbene **X**,^27^ which are shown in Figure 5. As expected, we indeed find similar polarization of the C−C σ‐bonds in compound **X**, for which the partial charges are 0.907 for the carbone carbon atom and 1.073 for the carbon atom of the NHC moiety. These partial charge distributions are the same for both IBOs of **X** depicted in Figure 5. The values are very close to those observed for **I**, and therefore, further support our description as a coordinative bond, rather than a regular C−C electron‐sharing bond. We note that the C−CH_3_ bonds of the Cp* moiety are also quite polarized towards the Cp ring, and that the C−C bonds within the Cp ring are deformed (see Figure S3, Supporting Information).

## Conclusion

Let us now reflect on our findings. On the basis of our calculations, we would describe the bonding in compound **I** as transition‐metal‐like with 1) coordination of an aromatic Cp*(−) ligand to the apical carbon atom, in which the ligand's π‐system is polarized toward the carbon owing to its high Lewis acidity; and 2) coordination from a lone pair located at the apical carbon atom toward a cationic CH_3_ group. The apical carbon atom therefore incorporates both possible modes of coordination chemistry at carbon, that is, serving as an electron‐pair donor and as an electron‐pair acceptor, all within a purely hydrocarbon framework.

We note here that for some mono‐ and dicationic organic molecules, a connection between the bonding models for organic and organometallic compounds was indicated by Hogeveen and Kwant in 1975,^28^ including compound **I**. This is particularly well reflected in the use of a coordination number for the apical carbon to account for the six bonding partners, rather than discussing how many “real” bonds are present.

The observation of a C(II) center, which coordinates to a cationic methyl group, can be considered as similar to the bonding in divalent C(0) compounds, which have been described as coordination compounds exhibiting dative bonding.^25^ Compound **I** therefore further extends the increasing number of compounds in which coordination chemistry at carbon has been observed,^29^ and reinforces the notion that main‐group elements can be teased into behaving like transition metals. Finally, we want to point out that compound **I**, which we have discussed in the present article in light of the potential of having six bonds, is markedly different from compounds such as CH_5_^+^,^30^ C(CH_3_)_5_^+^,^31^ or [C(Au(PPh_3_))_5_]^+^,^32^ which have been referred to as hypercoordinated compounds.^33^ For example, the bonding in CH_5_^+^ can be rationalized by invoking a three‐center two‐electron bonding interaction. It is the directionality of the bonding that differs between these hypercoordinated compounds, compound **I**, and, for example, the divalent C(0) compounds. In hypercoordinated compounds, the central carbon atom is formally reduced, and may be understood as an electron donor to its surrounding bonding partners. Considering the coordination compound [C(Au(PPh_3_))_5_]^+^, it becomes clear that the central carbon atom is donating *to* the Lewis‐acidic Au^I^ moieties. In compound **I**, however, the opposite is found with respect to the Cp* moiety. It is therefore the directionality of the bonding that sets these types of compounds apart.

## Conflict of interest

The authors declare no conflict of interest.

## Biographical Information

*Johannes E. M. N. Klein received his B.Sc. degree from the Universität Dortmund, Germany (renamed to Technische Universität Dortmund) and his M.Sc. from University College Dublin, Ireland in chemistry. Following research stays at Harvard University, USA (group of Prof. Dr. Tobias Ritter) and the University of York, UK (group of Prof. Dr. Richard J. K. Taylor) he obtained his doctorate degree from the Universität Stuttgart, Germany in 2014 under the tutelage of Prof. Dr. Bernd Plietker. After a postdoctoral stay at the University of Minnesota, USA in the group of Prof. Dr. Lawrence Que, Jr., he was in 2017 appointed as an assistant professor at the Stratingh Institute for Chemistry at the University of Groningen, The Netherlands. His research interests lie at the interface of organic, inorganic and computational chemistry*.

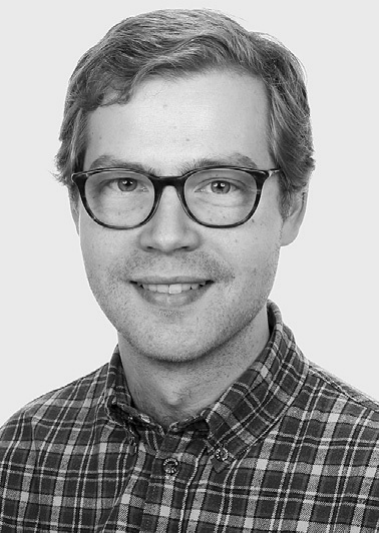

## Biographical Information

*After studying physics in Dresden from 2002 to 2006, Gerald Knizia switched field to theoretical chemistry. He obtained his Dr.rer.nat degree in Stuttgart in the group of Hans‐Joachim Werner, where he worked on high‐accuracy quantum chemistry methods (CCSD(T)‐F12) and the Molpro quantum chemistry package. After working on quantum embedding methods in the United States in the group of Garnet K.‐L. Chan at Cornell and Princeton, and a further stay in Stuttgart, Gerald Knizia has joined the chemistry department of the Pennsylvania State University in 2015 as an Assistant Professor. There he works on methods for the computational discovery and analysis of reaction mechanisms*.

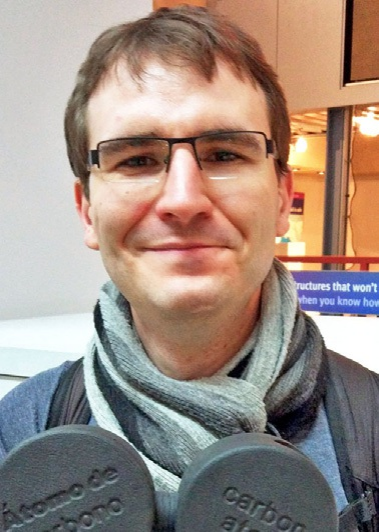

## Supporting information

As a service to our authors and readers, this journal provides supporting information supplied by the authors. Such materials are peer reviewed and may be re‐organized for online delivery, but are not copy‐edited or typeset. Technical support issues arising from supporting information (other than missing files) should be addressed to the authors.

Supplementary
