# Supplementary material for: The Pentagonal‐Pyramidal Hexamethylbenzene Dication: Many Shades of Coordination Chemistry at Carbon
Source: Chemistry. 2018 Mar 9;24(47):12340–5. doi: 10.1002/chem.201705812 (PMC6120489; doi:10.1002/chem.201705812)
Supplement: Supplementary file 1 — Supplementary [file CHEM-24-12340-s001.pdf]

CHEMISTRY
A European Journal

Supporting Information

The Pentagonal-Pyramidal Hexamethylbenzene Dication: Many Shades of Coordination Chemistry at Carbon

Johannes E. M. N. Klein,*[a] Remco W. A. Havenith,*[b, c] and Gerald Knizia*[d]

chem_201705812_sm_miscellaneous_information.pdf

Table of Contents

**1. Computational Details**	*S2-S3*
**2. Fractional Occupancy Density (FOD) Analysis of I - X**	*S3-S4*
**3. Intrinsic Bond Orbital (IBO) Depictions of I - IX**	*S5*
**4. IAO Partial Charge Analysis and C-C Bond Distances in Cp***	*S6-S7*
**5. Effective Oxidation States (EOS) Analysis of I - IX**	*S7*
**6. IBOs Associated with the C-C Bonds in the Cp* Fragment**	*S7*
**7. Cartesian Coordinates**	*S8-S13*
**8. References**	*S14-S15*

1. Computational Details

Geometries of were optimized at the TPSS[1]-D3(BJ)[2]/def2-TZVP[3] level of theory in the gas phase using Turbomole v7.0.1.[4] For Ru and Ir core electrons were replaced with a pseudo potential (ECP).[5] Calculations were accelerated using the MARI-J approach[6] employing Weigend's fitting basis sets.[7] Analytical second derivatives were computed to ensure that local minima had been reached showing no imaginary frequencies. Grid *m5* was used in all calculations. Starting geometries for **I** were based on coordinates reported in Ref [8] and for **VI** (CCDC 1294515)[9] on previously published crystallographic data.

Intrinsic bond orbital (IBO),[10] intrinsic atomic orbital (IAO) partial charge[10] and effective oxidation state (EOS)[11] analyses were carried out using IboView.[12] For IBO analysis the *iboexp* was set to 2 in all cases.

Fractional occupancy density (FOD)[13] analyses were carried out using *FODplot tools* provided by Grimme and co-workers.[14]

The current density induced by an external magnetic field was calculated with the B3LYP[15] functional and the def2-TZVP[3] basis set (for Ir with ECP),[5] using the ipsocentric CTOCD-DZ method,[16] as implemented in GAMESS-UK[17] and SYSMO.[18] The induced current density was plotted 1 a_0 below (in the opposite direction of the hexacoordinated carbon atom) the (median) plane formed by the carbon atoms forming the cyclopentadienyl ring. The applied field was directed perpendicular to the cyclopentadienyl ring. Anticlockwise circulations indicate paratropic currents (antiaromatic), while clockwise circulations indicate diatropic currents (aromatic). The ipsocentric approach allows a decomposition of the current density in orbital contributions,[16c, 19] and by visual inspection, the π-like orbitals have been selected to calculate the contribution of the π current density.

A summary of all studied Cp* compounds is shown in **Figure S1**.

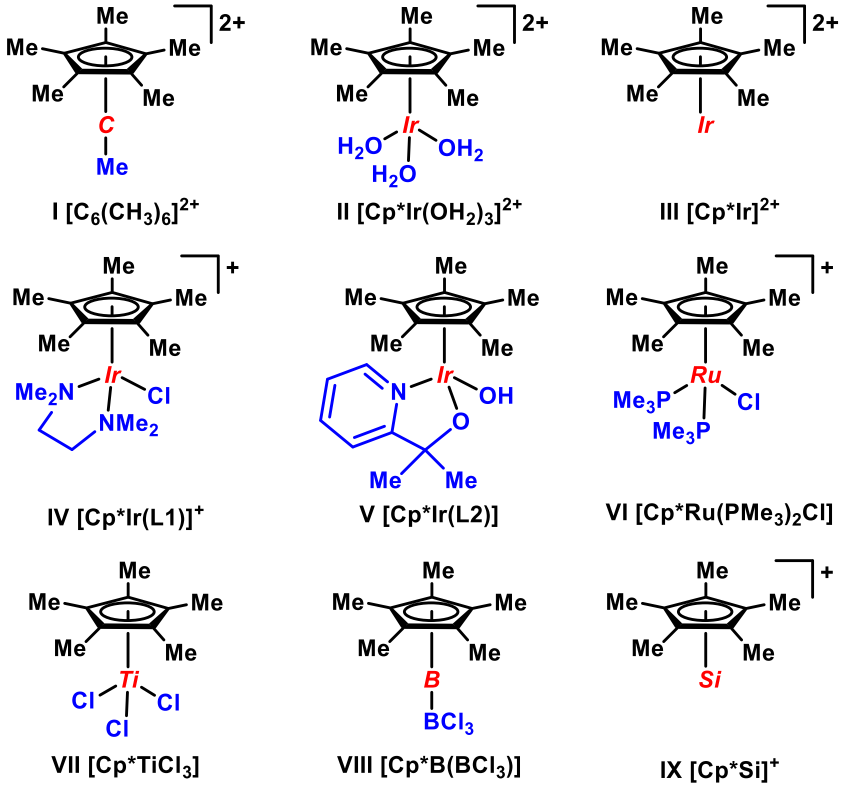

Figure S1: Summary of all studied Cp* compounds.
For representative articles or reviews see: ***C***,[8, 20] ***Ir***,[21] ***Ru***,[9] ***Ti***,[22] ***B***,[23] ***Si***.[24]

2. Fractional Occupancy Density (FOD) Analysis of I – X

FOD analyses were carried out at the TPSS-D3(BJ)/def2-TZVP level of theory at 5000 K as suggested by Grimme and co-workers.[13] Due to the low N_{FOD} values obtained, we show FOD plots using an isosurface value of 0.002 and not 0.005, the recommended default.

**Complex**	**N_{FOD}**	**FOD plot isosurface 0.002**
**I $C_6(CH_3)_6^{2+}$**	0.0035	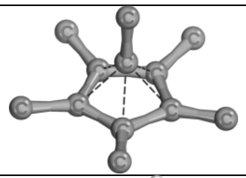
**II $[Cp^*Ir(OH_2)_3]^{2+}$**	0.1232	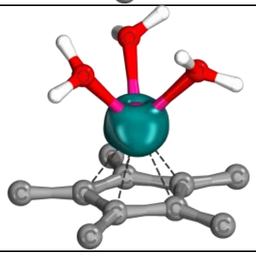

**III [Cp*Ir]$^{2+}$**	0.3130	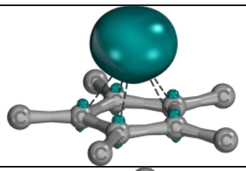
**IV [Cp*Ir(L1)]$^{2+}$**	0.1685	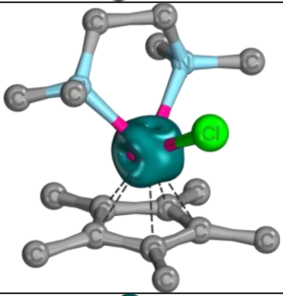
**V [Cp*Ir(L2)]$^{2+}$**	0.4036	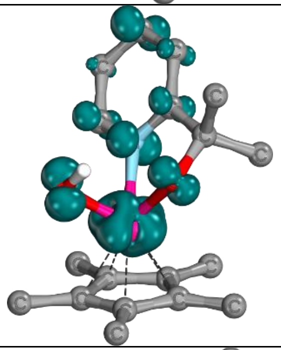
**VI [Cp*Ru(PCH$_3$)$_2$Cl]$^{+}$**	0.2140	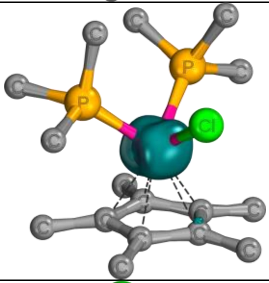
**VII [Cp*TiCl$_3$]**	0.3216	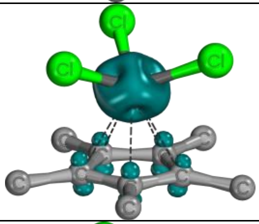
**VIII [Cp*B-BCl$_3$]**	0.0413	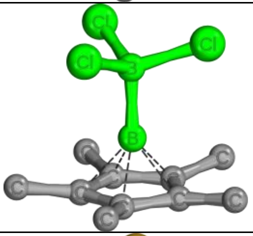
**IX [Cp*Si]$^{+}$**	0.0187	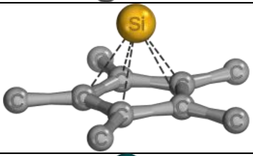
**X [(NHC)$_2$C]**	0.2089	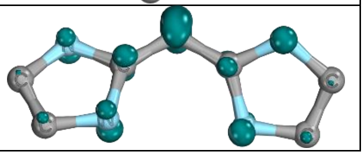

3. Intrinsic Bond Orbital (IBO) Depictions of I – IX

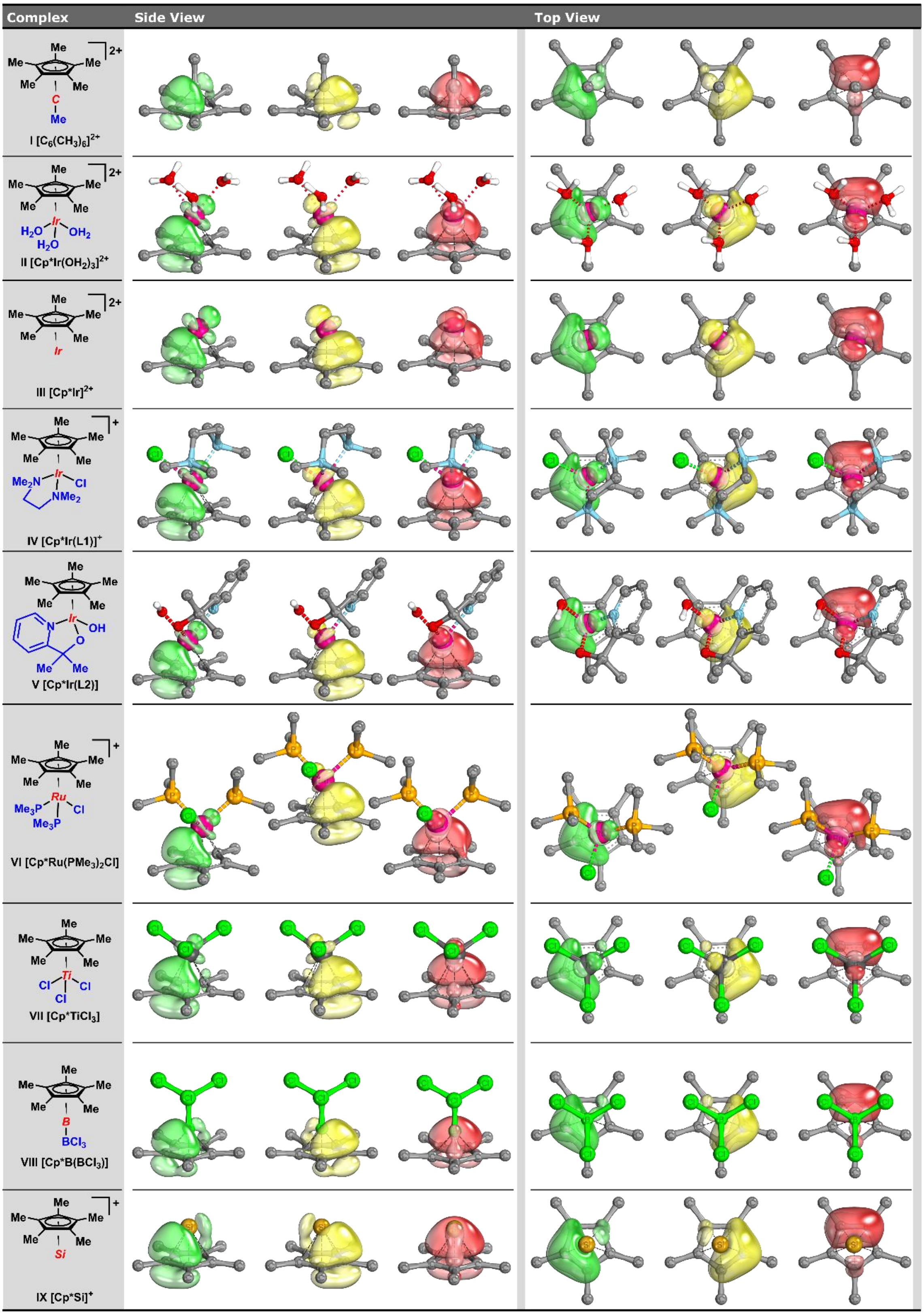

Figure S2: Intrinsic bond orbital (IBO) depictions of **I** – **IX**.

4. IAO Partial Charge Analysis and C-C Bond Distances in Cp*

A comparison of the intrinsic atomic orbital (IAO) partial charges of the Cp* ligand, the coordinated metal/element and ligands, where present, is less straightforward (see **Table 1**). For the $C_6(CH_3)_6^{2+}$ molecule the partial charge for the Cp* fragment surmounts to 1.563 and 0.437 for the CCH_3 fragment. At first glance this does seem to be inconsistent with the description of an anionic Cp* ligand on the apical carbon. However, for the additional complexes the partial charge for the Cp* fragment can be highly variable ranging from 1.251 to -0.195. Let us illustrate how easily the partial charge can be modulated through simple ligand variations in case of Cp*Ir-complexes, which do not affect the oxidation state of the Cp* fragment or the transition metal. For complex **II**, bearing three moderately Lewis-basic H_2O ligands, the partial charge of the Cp* ligand is 0.617 and for the Ir center 0.877. Upon removal of the H_2O ligands, we find a significantly increased partial charge of 1.251 for the Cp* fragment for complex **III**, which does not bear Lewis basic ligands on Ir. The opposite trend is observed if more donating/Lewis-basic ligands are introduced. For this purpose, we introduced complexes **IV** and **V** into our test set. Partial charges of 0.141 and -0.173 are observed for the Cp* fragments of **IV** and **V**, respectively. This leads to an ordering of **III** > **II** > **IV** > **V** concerning the partial charge, which spans a range from 1.251 to -0.173 for the Cp* fragment in this series of Ir complexes. This high level of variability of the partial charge does not alter the bonding interactions between the Cp* fragment and the Ir center qualitatively (compare depictions of IBOs in the ESI). As exemplified by the Ir complexes the partial charge can reach significantly positive values in this formally anionic ligand. As the role of the Cp* ligand in the series of Ir complexes is less debatable than in $C_6(CH_3)_6^{2+}$, we conclude that the partial charge is not a good measure to determine the best interpretation of the Cp* ligand—as previously found, partial charges are not a good device for interpreting formal bonding motives such as oxidation states.[25]

Complex	C-C Bond Distances in Cp* (Å)[a]	IAO Group Partial Charges		
		[Cp*]	[M]	[L]
I $C_6(CH_3)_6^{2+}$	1.451 (1.442)[b]	1.563	0.223	0.214
II $[Cp^*Ir(OH_2)_3]^{2+}$	1.453	0.617	0.877	0.505
III $[Cp^*Ir]^{2+}$	1.465	1.251	0.749	*n.a.*
IV $[Cp^*Ir(L1)]^{+}$	1.447	0.141	0.841	-0.123
V $[Cp^*Ir(L2)]$	1.449	-0.173	1.057	-0.884
VI $[Cp^*Ru(PCH_3)_2Cl]^{+}$	1.441	-0.195	0.363	-0.168
VII $[Cp^*TiCl_3]$	1.428	0.018	1.079	-1.097
VIII $[Cp^*B\text{-}BCl_3]$	1.436	0.594	0.180	-0.775
IX $[Cp^*Si]^{+}$	1.441	0.421	0.579	*n.a.*

[a] Averaged computed C-C bond distance over all five C-C bonds in the Cp* carbocycle.
[b] Experimentally determined distance based on structural parameters in Ref [8].

5. Effective Oxidation States (EOS) Analysis of I – IX

Complex	Ox. State Cp*	Ox. State [M]	Ox. State [L][a]	*R* (%)[b]
I $C_6(CH_3)_6^{2+}$	-I	+II	+I	63.17[61.77]
II $[Cp^*Ir(OH_2)_3]^{2+}$	-I	+III	0	61.30[53.24]
III $[Cp^*Ir]^{2+}$	-I	+III	*n.a.*	57.63[65.34]
IV $[Cp^*Ir(L1)]^{2+}$	-I	+III	-I	70.28[62.75]
V $[Cp^*Ir(L2)]^{2+}$	-I	+III	-II	78.09[70.15]
VI $[Cp^*Ru(PCH_3)_2Cl]^{+}$	-I	+II	-I	77.77[70.87]
VII $[Cp^*TiCl_3]$	-I	+IV	-III	100.00[91.90]
VIII $[Cp^*B\text{-}BCl_3]$	-I	+I	0	80.67[61.51]
IX $[Cp^*Si]^{+}$	-I	+II	*n.a.*	100.00[99.99]

[a] Group oxidation state for all ligands (except Cp*) bound to the [M] fragment. [b] Formal assignment reliability based on topological fuzzy Voronoi cells (TFVC) and based on intrinsic atomic orbitals (IAO). The latter values are given in brackets.

6. IBOs Associated with the C-C Bonds in the Cp* Fragment

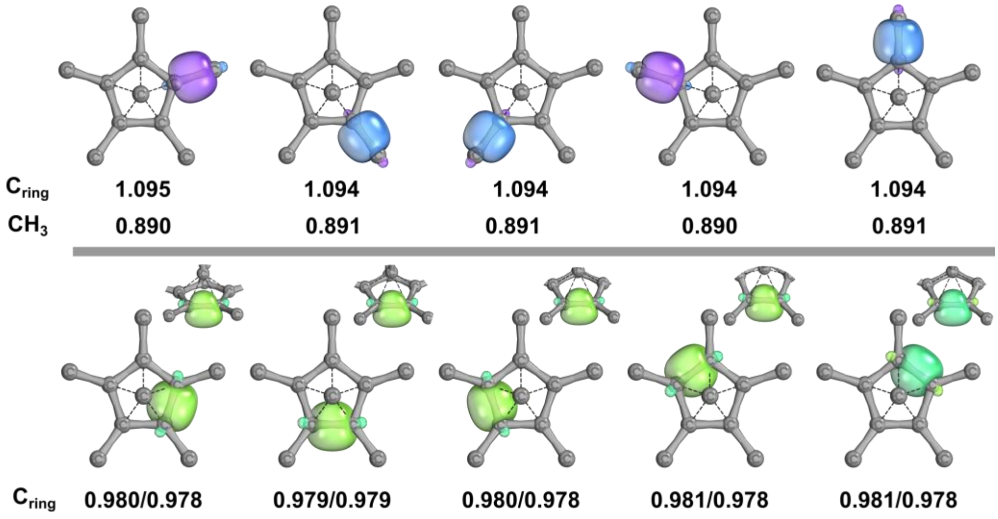

Figure S3: IBOs associated with the C-C bonds in the Cp* fragment. Values listed correspond to the IAO partial charge distribution between the two carbon atoms of the depicted IBOs.

7. Cartesian Coordinates

C -0.6425232 -1.0541912 -0.4637623<br>C 0.8031343 -0.9378464 -0.4643341<br>C 1.1391753 0.4748300 -0.4598032<br>C -0.0991959 1.2313286 -0.4658742<br>C -1.2004369 0.2866051 -0.4604347<br>C -2.6386218 0.6277489 -0.2670376<br>C -0.2174586 2.7033179 -0.2708538<br>C 2.5041768 1.0420380 -0.2663044<br>C 1.7657785 -2.0583343 -0.2702474<br>C -0.0000690 -0.0000610 0.7158528<br>C -1.4136535 -2.3144102 -0.2700146<br>C -0.0001681 -0.0002025 2.2058345<br>H -3.0818628 0.7245019 -1.2672451<br>H -2.7697008 1.5784091 0.2514906<br>H -3.1794047 -0.1569996 0.2637853<br>H -1.1359062 2.9771914 0.2502100<br>H -0.2507931 3.1558641 -1.2713678<br>H 0.6430818 3.1194809 0.2549317<br>H 2.4812264 2.0007274 0.2535346<br>H 2.9256648 1.2101515 -1.2664497<br>H 3.1640385 0.3535858 0.2634190<br>H 1.3125118 -2.9059195 0.2455217<br>H 2.6641410 -1.7413863 0.2614653<br>H 2.0693782 -2.3952860 -1.2707377<br>H -2.3510013 -2.1453702 0.2619425<br>H -0.8306805 -3.0786282 0.2457231<br>H -1.6595292 -2.6953574 -1.2705323<br>H -0.8679134 -0.5527889 2.5777388<br>H -0.0449375 1.0278280 2.5773181<br>H 0.9123501 -0.4752588 2.5777389	**I $C_6(CH_3)_6^{2+}$**<br><br>**$E_{TPSS-D3(BJ)/def2-TZVP}$: -467.69836061367**
Ir 0.4309347 -0.0065458 -0.7487613<br>C 0.3478440 0.8433473 1.2136919<br>C -0.8843179 1.1337848 0.5155284<br>C -1.5084364 -0.1238586 0.1362655<br>C -0.6730500 -1.1991297 0.6586930<br>C 0.4735827 -0.6112962 1.3086196<br>C 1.2731199 1.8431338 1.8245637<br>C -1.4282847 2.4929060 0.2332379<br>C -2.8352235 -0.2834909 -0.5313134<br>C -0.9684848 -2.6571492 0.5569867<br>C 1.5549913 -1.3493691 2.0252095<br>H -1.5466565 -2.9543919 1.4422296<br>H -0.0589884 -3.2614232 0.5417414<br>H -1.5794316 -2.8926546 -0.3176506<br>H 1.7292497 -2.3390480 1.5978136<br>H 2.4937831 -0.7905568 2.0367973<br>H 1.2503056 -1.4890705 3.0707820<br>H 1.2616599 2.7930347 1.2862954<br>H 0.9486985 2.0402187 2.8546194<br>H 2.2999958 1.4742086 1.8713032<br>H -2.0719564 2.7875706 1.0731208<br>H -2.0482075 2.5086307 -0.6663588<br>H -0.6388984 3.2439776 0.1529577<br>H -2.9004295 -1.2193338 -1.0904680<br>H -3.6198934 -0.3023212 0.2358896<br>H -3.0534056 0.5457867 -1.2073375<br>O 2.5942224 -0.0269472 -1.3488043<br>O 0.5665913 -1.3702317 -2.5174369	**II $[Cp^*Ir(OH_2)_3]^{2+}$**<br><br>**$E_{TPSS-D3(BJ)/def2-TZVP}$: -723.64841595380**

O	0.5688506	1.3324307	-2.5290233	
H	1.4659862	-1.7139229	-2.6805599	
H	-0.0458113	-2.1233576	-2.5916919	
H	2.8784745	0.7541851	-1.8607443	
H	3.2777631	-0.1918486	-0.6755948	
H	0.3075131	0.9076543	-3.3685807	
H	0.1379103	2.2050769	-2.5020130	
Ir	0.4087564	-0.0070240	-0.7097490	**III [Cp*Ir]$^{2+}$**
C	0.3396040	0.8459800	1.2480581	
C	-0.8992799	1.1459559	0.5253515	**$E_{TPSS-D3(BJ)/def2-TZVP}$: -494.06500406510**
C	-1.5350813	-0.1253904	0.1694819	
C	-0.6891961	-1.2110833	0.6723930	
C	0.4694216	-0.6107385	1.3389964	
C	1.2812853	1.8417066	1.8139826	
C	-1.4311423	2.4985105	0.2318030	
C	-2.8230559	-0.2850204	-0.5475301	
C	-0.9712719	-2.6618819	0.5535844	
C	1.5654285	-1.3474596	2.0131432	
H	-1.5593859	-2.9571326	1.4383178	
H	-0.0602262	-3.2639695	0.5540776	
H	-1.5710351	-2.8979040	-0.3279547	
H	1.7267627	-2.3380145	1.5827061	
H	2.5028040	-0.7872421	2.0156718	
H	1.2675984	-1.4919928	3.0649191	
H	1.2698561	2.7846006	1.2632329	
H	0.9516251	2.0596580	2.8433635	
H	2.3027473	1.4602730	1.8745919	
H	-2.0706051	2.7928487	1.0804921	
H	-2.0544404	2.5145816	-0.6647231	
H	-0.6403060	3.2467745	0.1468792	
H	-2.8753547	-1.2229843	-1.1043297	
H	-3.6223794	-0.3099285	0.2116016	
H	-3.0346288	0.5538370	-1.2139046	
Ir	0.5740698	0.0600819	-0.7297874	**IV [Cp*Ir(L1)]$^{2+}$**
C	0.1717273	1.1085580	1.1059778	
C	-1.0506099	1.0601728	0.3091621	**$E_{TPSS-D3(BJ)/def2-TZVP}$: -1302.86758408726**
C	-1.4103120	-0.3134592	0.1297370	
C	-0.4227078	-1.1471083	0.8046490	
C	0.5205598	-0.2510706	1.4355237	
C	0.8404758	2.3535207	1.5946635	
C	-1.8238691	2.2453983	-0.1678038	
C	-2.6863746	-0.7809143	-0.4983478	
C	-0.5162734	-2.6233135	1.0359489	
C	1.5912444	-0.6525943	2.3982934	
H	-1.0477211	-2.8259659	1.9742982	
H	0.4729336	-3.0829260	1.1101071	
H	-1.0658285	-3.1227149	0.2350562	
H	1.8754373	-1.7005407	2.2866415	
H	2.4824926	-0.0257971	2.3155013	
H	1.2027422	-0.5255804	3.4166768	
H	0.8490139	3.1186503	0.8150172	
H	0.3054342	2.7514637	2.4654706	
H	1.8729864	2.1597204	1.8949788	
H	-2.4797724	2.5959725	0.6390843	
H	-2.4477317	1.9958888	-1.0286647	
H	-1.1573863	3.0608263	-0.4535685	
H	-2.6814018	-1.8552760	-0.6897106	
H	-3.5139713	-0.5769875	0.1919439	
H	-2.9037968	-0.2532579	-1.4307654	

N	2.6954470	-0.4055681	-1.1334801
N	0.4222221	-1.0278840	-2.6946142
Cl	1.0137138	2.0790647	-1.9675524
C	1.7973899	-1.4062180	-3.1819801
C	2.8399849	-0.4676874	-2.6225909
H	2.7013621	0.5475846	-2.9966191
H	3.8490969	-0.8165585	-2.8773698
H	1.9911199	-2.4330202	-2.8643027
H	1.8115386	-1.3925985	-4.2768160
C	3.0952925	-1.7077727	-0.5288062
C	3.6180533	0.6480462	-0.6143869
C	-0.2579255	-0.1949839	-3.7360311
C	-0.3604066	-2.2870850	-2.5526280
H	-1.3901530	-2.0401306	-2.3045505
H	0.0772782	-2.8957604	-1.7609368
H	-0.3456746	-2.8431657	-3.4983190
H	4.6503094	0.3943469	-0.8850231
H	3.5274570	0.6876580	0.4713816
H	3.3313136	1.6059163	-1.0460736
H	2.3826907	-2.4857112	-0.8008186
H	4.0997832	-1.9854906	-0.8724522
H	3.1068055	-1.5994806	0.5533019
H	-0.3935556	-0.7983966	-4.6419242
H	-1.2274593	0.1229251	-3.3513691
H	0.3416433	0.6879854	-3.9448783

V [Cp*Ir(L2)]$^{2+}$

$E_{TPSS-D3(BJ)/def2-TZVP}$: **-1011.78889282415**

Ir	0.2247246	0.0631379	-0.6061182
C	-0.2012396	1.1377403	1.2343690
C	-1.4508134	0.9645193	0.4910668
C	-1.6592209	-0.4451270	0.3264722
C	-0.5551942	-1.1590762	0.9562509
C	0.3229704	-0.1619064	1.5442847
C	0.3471251	2.4523032	1.6982291
C	-2.3738749	2.0714013	0.0831317
C	-2.7993614	-1.1113858	-0.3780749
C	-0.4019323	-2.6441408	1.0719433
C	1.6049424	-0.4746900	2.2509045
H	-0.8485792	-3.0185206	2.0032420
H	0.6557184	-2.9208387	1.0625266
H	-0.8878860	-3.1511353	0.2340529
H	2.2552531	-1.0559008	1.5876973
H	2.1353920	0.4383317	2.5322437
H	1.4103905	-1.0538093	3.1615671
H	0.1426816	3.2459674	0.9731573
H	-0.1149569	2.7494177	2.6490568
H	1.4283125	2.4000998	1.8532807
H	-2.9885383	2.4015180	0.9318109
H	-3.0500643	1.7510354	-0.7136523
H	-1.8160037	2.9398283	-0.2808279
H	-2.4268558	-1.8668314	-1.0759729
H	-3.4596275	-1.6032509	0.3471372
H	-3.3920839	-0.3911482	-0.9462152
O	2.1081503	-0.6767756	-0.8453404
O	-0.0397053	-0.9181480	-2.4062657
N	0.8715945	1.4669660	-2.0141665
C	0.5761140	1.1964420	-3.3089920
C	1.0222494	2.0404444	-4.3246797
C	1.7851809	3.1618064	-4.0123101
C	2.1034073	3.4080215	-2.6767786
C	1.6376147	2.5321796	-1.7083398
C	-0.2644374	-0.0562099	-3.4966265

H 0.7740388 1.8090902 -5.3549689<br>H 2.1376206 3.8246567 -4.7971335<br>H 2.7164977 4.2542235 -2.3855227<br>H 1.8764843 2.6510598 -0.6587429<br>C -1.7490540 0.3779471 -3.5690211<br>H -1.9416332 1.0375572 -4.4233367<br>H -2.3726457 -0.5167144 -3.6579480<br>H -2.0157141 0.9051614 -2.6482440<br>C 0.1284677 -0.8176782 -4.7718631<br>H -0.4480962 -1.7461767 -4.8034087<br>H -0.0872370 -0.2456984 -5.6816936<br>H 1.1923966 -1.0693201 -4.7472187<br>H 2.0218190 -1.1744399 -1.6805827	
Ru -0.3258769 0.2650236 -0.1108953<br>Cl -1.5605097 2.2596373 0.5989510<br>P 1.4525281 1.3738658 0.7971558<br>P -0.7778033 -0.7659348 1.8795520<br>C -1.4466124 -1.1109993 -1.5077363<br>C -1.5389822 0.2216283 -1.9954828<br>C -0.2117037 0.7146802 -2.2767918<br>C 0.7125155 -0.3395956 -1.9392783<br>C -0.0380158 -1.4583227 -1.4247170<br>C -2.6060555 -2.0470297 -1.3434803<br>C -2.8012345 0.9821678 -2.2514963<br>C 0.0793220 2.0149396 -2.9623181<br>C 2.1704131 -0.3668174 -2.2838629<br>C 0.5189415 -2.8192456 -1.1312591<br>C 1.9420760 2.8757019 -0.1472992<br>C 3.0866974 0.5329080 0.9831025<br>C 1.2932181 2.1318170 2.4691693<br>C -1.9260862 -2.2116256 1.8725985<br>C -1.6001459 0.2111238 3.2074743<br>C 0.6084381 -1.5546314 2.8181828<br>H -2.3401347 -2.9265014 -0.7548951<br>H -2.9377399 -2.4012358 -2.3292704<br>H -3.4601981 -1.5603310 -0.8649418<br>H -2.7057565 2.0152882 -1.9076892<br>H -3.6482447 0.5334739 -1.7272320<br>H -3.0265516 0.9876498 -3.3266861<br>H -0.2735407 1.9952040 -4.0026807<br>H 1.1513720 2.2257624 -2.9840858<br>H -0.4213401 2.8452949 -2.4543263<br>H 2.6320462 0.6209730 -2.1993388<br>H 2.2997738 -0.7008180 -3.3223382<br>H 2.7211655 -1.0602619 -1.6435298<br>H -0.0943390 -3.3532249 -0.3997884<br>H 1.5332286 -2.7514535 -0.7278807<br>H 0.5586437 -3.4365441 -2.0402370<br>H 2.2947924 2.5962274 -1.1424780<br>H 2.7325352 3.4305326 0.3701418<br>H 1.0533970 3.5040086 -0.2520581<br>H 2.9787519 -0.3498967 1.6176817<br>H 3.8215658 1.2108399 1.4319067<br>H 3.4542056 0.2093661 0.0075513<br>H 2.1614413 2.7653129 2.6799000<br>H 1.2274393 1.3606722 3.2404290<br>H 0.3800396 2.7316062 2.4821500<br>H -2.0787980 -2.5687009 2.8970007<br>H -1.5125011 -3.0275333 1.2753546<br>H -2.8890995 -1.9187948 1.4492659	**VI [Cp*Ru(PCH$_3$)$_2$Cl]$^+$**<br><br>**$E_{TPSS-D3(BJ)/def2-TZVP}$: -1868.13358000391**

H -2.5613986 0.5641652 2.8267134<br>H -1.0101625 1.0925012 3.4591781<br>H -1.7503654 -0.4055245 4.1008936<br>H 0.2389188 -2.0835366 3.7041906<br>H 1.3320706 -0.8006822 3.1368040<br>H 1.1209433 -2.2650775 2.1625850	
Ti 0.3897370 -0.0104866 -0.6867012<br>C 0.0163032 0.9742733 1.4203633<br>C -1.2236130 1.0194804 0.7099898<br>C -1.6483773 -0.3215182 0.4802601<br>C -0.6724150 -1.1975322 1.0497633<br>C 0.3425125 -0.3956480 1.6526067<br>C 0.8164268 2.1574346 1.8738921<br>C -1.9890292 2.2548270 0.3498550<br>C -2.9345536 -0.7347882 -0.1653169<br>C -0.7241904 -2.6951584 1.0486505<br>C 1.4875113 -0.8990368 2.4737609<br>H -1.2448974 -3.0598547 1.9436908<br>H 0.2801482 -3.1269941 1.0470185<br>H -1.2549082 -3.0702256 0.1704937<br>H 1.8197512 -1.8840459 2.1389197<br>H 2.3437818 -0.2228821 2.4261285<br>H 1.1759516 -0.9823266 3.5233557<br>H 0.6743988 3.0089319 1.2042580<br>H 0.5097817 2.4622217 2.8829931<br>H 1.8843815 1.9256927 1.9046163<br>H -2.6946717 2.5010919 1.1541964<br>H -2.5627490 2.1181823 -0.5701772<br>H -1.3232502 3.1082004 0.2072824<br>H -2.8503439 -1.7220372 -0.6236774<br>H -3.7322435 -0.7734616 0.5881532<br>H -3.2396561 -0.0282582 -0.9412618<br>Cl 2.5155102 -0.6900613 -0.5143390<br>Cl -0.4818112 -1.3623001 -2.2475163<br>Cl 0.5438312 1.9844172 -1.6961851	**VII [Cp*TiCl$_3$]**<br><br>**$E_{TPSS-D3(BJ)/def2-TZVP}$: -2620.85752288591**
C -0.6363096 -1.0424743 -0.5186413<br>C 0.7913342 -0.9299846 -0.5188449<br>C 1.1260490 0.4682537 -0.5042435<br>C -0.0964338 1.2213034 -0.5174877<br>C -1.1859362 0.2861198 -0.5039397<br>C -2.6372026 0.5983512 -0.3036077<br>C -0.2126977 2.7051345 -0.3629232<br>C 2.5104257 1.0042961 -0.3042894<br>C 1.7845543 -2.0366731 -0.3410565<br>B -0.0006063 0.0063883 0.7501529<br>C -1.4438468 -2.2911430 -0.3409737<br>B -0.0008797 0.0169921 2.4304041<br>H -3.1889024 0.4234014 -1.2335656<br>H -2.7790679 1.6390644 -0.0088953<br>H -3.0580344 -0.0380597 0.4791206<br>H -1.1204294 2.9736561 0.1788070<br>H -0.2406842 3.1727690 -1.3536988<br>H 0.6337107 3.1094410 0.1936596<br>H 2.4872826 2.0550638 -0.0120795<br>H 3.0832777 0.9157349 -1.2336908<br>H 3.0252606 0.4434000 0.4800353<br>H 1.2910108 -3.0093537 -0.3201866<br>H 2.3271778 -1.9095265 0.6004848<br>H 2.5017326 -2.0278455 -1.1679319<br>H -1.9994985 -2.2509266 0.6006589	**VIII [Cp*B-BCl$_3$]**<br><br>**$E_{TPSS-D3(BJ)/def2-TZVP}$: -<br>1820.91499555370**

H -0.8039395 -3.1744457 -0.3205026<br>H -2.1536596 -2.3945595 -1.1677805<br>Cl -1.4745635 -0.9873382 3.0432701<br>Cl -0.1416730 1.7972010 3.0175587<br>Cl 1.6133469 -0.7425379 3.0416971	
C -0.6384247 -1.0460813 -0.2846019<br>C 0.7977264 -0.9304301 -0.2843945<br>C 1.1315534 0.4711828 -0.2840723<br>C -0.0982994 1.2217916 -0.2841110<br>C -1.1922115 0.2840570 -0.2843751<br>C -2.6492662 0.6310517 -0.3200538<br>C -0.2185972 2.7147406 -0.3193132<br>C 2.5143729 1.0467533 -0.3193969<br>C 1.7725533 -2.0675607 -0.3200312<br>Si -0.0001679 -0.0002210 1.4804113<br>C -1.4187184 -2.3245484 -0.3204831<br>H -2.9728214 0.7079437 -1.3646452<br>H -2.8482670 1.5901944 0.1621798<br>H -3.2592232 -0.1352721 0.1624532<br>H -1.1360545 3.0577693 0.1630753<br>H -0.2452184 3.0465744 -1.3637928<br>H 0.6319545 3.2002876 0.1632811<br>H 2.5571479 2.0257132 0.1621737<br>H 2.8219975 1.1736687 -1.3639168<br>H 3.2387613 0.3881551 0.1639306<br>H 1.3696073 -2.9606084 0.1618379<br>H 2.7162769 -1.8061923 0.1628293<br>H 1.9894414 -2.3196942 -1.3646157<br>H -2.3922691 -2.2175584 0.1620783<br>H -0.8782138 -3.1416064 0.1615475<br>H -1.5921703 -2.6081197 -1.3651168	**IX $[Cp^*Si]^+$**<br><br>**$E_{TPSS-D3(BJ)/def2-TZVP}$: -679.64466039733**
N -1.1145284 0.3586213 -2.0100854<br>C -0.0465339 0.9154672 -1.2683956<br>N -0.1080164 2.2573202 -1.6417442<br>C -1.2128902 2.5288537 -2.4393598<br>C -1.8533670 1.3557027 -2.6541530<br>C 0.8053495 0.3779329 -0.3464056<br>C 1.0529099 -0.9646452 -0.3167459<br>N 1.5889341 -1.6747332 0.7566702<br>C 1.9186342 -2.9790832 0.4092907<br>C 1.5413185 -3.1612811 -0.8779764<br>N 0.9480512 -1.9698822 -1.3063601<br>H 1.8553156 -1.1921978 1.6013764<br>H -1.4884193 -0.5464324 -1.7632558<br>H 0.4964424 2.9343108 -1.2016980<br>H 0.8314140 -1.7181851 -2.2773912<br>H 2.3585332 -3.6693807 1.1108398<br>H -2.7339011 1.1378196 -3.2376634<br>H -1.4280219 3.5181689 -2.8096969<br>H 1.6013776 -4.0362345 -1.5057947	**X $[(NHC)_2C]$**<br><br>**$E_{TPSS-D3(BJ)/def2-TZVP}$: -490.76418085282**

8. References

[1] J. Tao, J. P. Perdew, V. N. Staroverov, G. E. Scuseria, *Phys. Rev. Lett.* **2003**, *91*, 146401.

[2] (a) S. Grimme, J. Antony, S. Ehrlich, H. Krieg, *J. Chem. Phys.* **2010**, *132*, 154104; (b) S. Grimme, S. Ehrlich, L. Goerigk, *J. Comput. Chem.* **2011**, *32*, 1456-1465.

[3] F. Weigend, R. Ahlrichs, *Phys. Chem. Chem. Phys.* **2005**, *7*, 3297-3305.

[4] (a) R. Ahlrichs, M. Bär, M. Häser, H. Horn, C. Kölmel, *Chem. Phys. Lett.* **1989**, *162*, 165-169; (b) F. Furche, R. Ahlrichs, C. Hättig, W. Klopper, M. Sierka, F. Weigend, *WIREs Comput. Mol. Sci.* **2014**, *4*, 91-100; (c) *TURBOMOLE V7.0.1 2015, a development of University of Karlsruhe and Forschungszentrum Karlsruhe GmbH, 1989-2007, TURBOMOLE GmbH, since 2007; available from* http://www.turbomole.com.

[5] D. Andrae, U. Häußermann, M. Dolg, H. Stoll, H. Preuß, *Theor. Chim. Acta* **1990**, *77*, 123-141.

[6] M. Sierka, A. Hogekamp, R. Ahlrichs, *J. Chem. Phys.* **2003**, *118*, 9136-9148.

[7] F. Weigend, *Phys. Chem. Chem. Phys.* **2006**, *8*, 1057-1065.

[8] M. Malischewski, K. Seppelt, *Angew. Chem. Int. Ed.* **2017**, *56*, 368-370.

[9] D. C. Smith, C. M. Haar, L. Luo, C. Li, M. E. Cucullu, C. H. Mahler, S. P. Nolan, W. J. Marshall, N. L. Jones, P. J. Fagan, *Organometallics* **1999**, *18*, 2357-2361.

[10] G. Knizia, *J. Chem. Theory Comput.* **2013**, *9*, 4834-4843.

[11] E. Ramos-Cordoba, V. Postils, P. Salvador, *J. Chem. Theory Comput.* **2015**, *11*, 1501-1508.

[12] (a) G. Knizia, http://www.iboview.org/; (b) G. Knizia, J. E. M. N. Klein, *Angew. Chem. Int. Ed.* **2015**, *54*, 5518-5522.

[13] (a) S. Grimme, A. Hansen, *Angew. Chem. Int. Ed.* **2015**, *54*, 12308-12313; (b) C. A. Bauer, A. Hansen, S. Grimme, *Chemistry* **2017**, *23*, 6150-6164.

[14] *FODplot tools for TURBOMOLE (6.7 or higher),* http://www.thch.uni-bonn.de/tc/index.php?section=downloads&subsection=FODplot&lang=english*, (accessed 02/27/2017)*.

[15] (a) A. D. Becke, *J. Chem. Phys.* **1993**, *98*, 5648-5652; (b) A. D. Becke, *Phys. Rev. A* **1988**, *38*, 3098-3100; (c) C. Lee, W. Yang, R. G. Parr, *Phys. Rev. B* **1988**, *37*, 785-789.

[16] (a) T. A. Keith, R. F. W. Bader, *Chem. Phys. Lett.* **1993**, *210*, 223-231; (b) S. Coriani, P. Lazzeretti, M. Malagoli, R. Zanasi, *Theor. Chim. Acta* **1994**, *89*, 181-192; (c) E. Steiner, P. W. Fowler, *J. Phys. Chem. A* **2001**, *105*, 9553-9562.

[17] (a) M. F. Guest, I. J. Bush, H. J. J. Van Dam, P. Sherwood, J. M. H. Thomas, J. H. Van Lenthe, R. W. A. Havenith, J. Kendrick, *Mol. Phys.* **2005**, *103*, 719-747; (b) R. W. A. Havenith, P. W. Fowler, *Chem. Phys. Lett.* **2007**, *449*, 347-353.

[18] *P. Lazzeretti, R. Zanasi, Sysmo package (University of Modena), 1980. Additional routines by P. W. Fowler, E. Steiner, R. W. A. Havenith, A. Soncini.*

[19] (a) E. Steiner, P. W. Fowler, *Chem. Commun.* **2001**, 2220-2221; (b) E. Steiner, P. W. Fowler, *Phys. Chem. Chem. Phys.* **2004**, *6*, 261-272.

[20] H. Hogeveen, P. W. Kwant, *Tetrahedron Lett.* **1973**, *14*, 1665-1670.

[21] J. M. Thomsen, D. L. Huang, R. H. Crabtree, G. W. Brudvig, *Dalton Trans.* **2015**, *44*, 12452-12472.

[22] Y. Zhang, Y. Mu, *Organometallics* **2006**, *25*, 631-634.
[23] P. Greiwe, A. Bethäuser, H. Pritzkow, T. Kühler, P. Jutzi, W. Siebert, *Eur. J. Inorg. Chem.* **2000**, *2000*, 1927-1929.
[24] P. Jutzi, *Chem. Eur. J.* **2014**, *20*, 9192-9207.
[25] (a) G. Aullón, S. Alvarez, *Theor. Chem. Acc.* **2009**, *123*, 67-73; (b) M. Kaupp, H. G. von Schnering, *Angew. Chem. Int. Ed.* **1995**, *34*, 986-986; (c) R. Hoffmann, S. Alvarez, C. Mealli, A. Falceto, T. J. Cahill, T. Zeng, G. Manca, *Chem. Rev.* **2016**, *116*, 8173-8192; (d) H. Raebiger, S. Lany, A. Zunger, *Nature* **2008**, *453*, 763-766; (e) M. Jansen, U. Wedig, *Angew. Chem. Int. Ed.* **2008**, *47*, 10026-10029.
